# RECQL5 plays an essential role in maintaining genome stability and viability of triple‐negative breast cancer cells

**DOI:** 10.1002/cam4.2349

**Published:** 2019-06-23

**Authors:** Jin Peng, Lichun Tang, Mengjiao Cai, Huan Chen, Jiemin Wong, Pumin Zhang

**Affiliations:** ^1^ Shanghai Key Laboratory of Regulatory Biology, Institute of Biomedical Sciences, School of Life Sciences East China Normal University Shanghai China; ^2^ State Key Laboratory of Proteomics, National Center for Protein Sciences (Beijing), Beijing Proteome Research Center Beijing Institute of Lifeomics Beijing China; ^3^ Department of Oncology, The First Affiliated Hospital Xi'an Jiaotong University Medical College Xi'an China

**Keywords:** DNA damage, essential gene, genomic stability, RECQL5, triple‐negative breast cancer

## Abstract

Triple‐negative breast cancer (TNBC) is a malignancy that currently lacks targeted therapies. The majority of TNBCs can be characterized as basal‐like and has an expression profile enriched with genes involved in DNA damage repair and checkpoint response. Here, we report that TNBC cells are under replication stress and are constantly generating DNA double‐strand breaks, which is not seen in non‐TNBC cells. Consequently, we found that *RECQL5*, which encodes a RecQ family DNA helicase involved in many aspects of DNA metabolism including replication and repair, was essential for TNBC cells to survive and proliferate in vitro and in vivo. Compromising RECQL5 function in TNBC cells results in persistence of DNA damage, G2 arrest, and ultimately, cessation of proliferation. Our results suggest RECQL5 may be a potential therapeutic target for TNBC.

## 1 INTRODUCTION

Genome instability, resulted from defects in DNA metabolism including replication and repair, is a hallmark of cancer.^1^ These defects are caused by mutations in genes involved in maintaining the integrity of the genome. For example, the loss of function mutations in *BRCA1* and *BRCA2*, two genes important for the repair of double‐strand breaks (DSBs) through homologous recombination (HR), accounts for a large fraction of hereditary breast and ovarian cancers.^2^ On the other hand, genome instability has also been exploited for cancer therapy with the idea that additional instability brought about by chemotherapy agents or radiation would push cancer cells into death or senescence due to the accumulation of excessive DNA damages.

About 10 to 20% of breast cancer are triple‐negative for lacking significant expression of estrogen receptor (ER), progesterone receptor (PR), and human epidermal growth factor receptor 2 (HER2).^3^ Triple‐negative breast cancer (TNBC) is more aggressive and has poorer prognosis than other breast malignancies. The majority of TNBCs can be characterized as basal‐like^4^ and have an expression profile enriched with genes involved in DNA damage checkpoint response.^5^, ^6^ More than 80% of breast cancer patients with a hereditary *BRCA1* mutation are assigned to TNBC subtype.^7^ Some sporadic TNBCs also show similar characteristics with *BRCA1*‐mutant tumors. Together, they are referred as BRCAness.^8^ While targeted therapies are available for other subtypes of breast cancer, TNBCs currently lack targeted therapies (except *BRCA1* mutated ones) and their treatment relies heavily on broad cytotoxic chemotherapeutic agents.^9^

RecQ family of DNA helicases includes *RECQL1*, Bloom syndrome gene (*BLM*), Werner syndrome gene (*WRN*), *RECQL4*, and *RECQL5*. They play overlapping as well as unique functions in DNA replication and damage (especially double‐strand breaks) repair.^10^ Bloom and Werner syndromes are characterized with premature aging and predisposition to cancer.^11^ Mutations in *RECQL4* are associated with Rothmund‐Thomson syndrome (RTS), another premature aging and cancer predisposition disease.^11^ More recently, mutations in *RECQL1* were found in familial breast cancer patients.^12^, ^13^

Although no specific human diseases have been linked to loss of RECQL5 function, this helicase plays important roles in DNA metabolism as other members of the family do^14^ and in relieving transcription‐induced chromosomal stress uniquely.^15^, ^16^ *Recql5*‐deficient mice are viable but display increased levels of sister‐chromatid exchange and are predisposed to several types of cancers, at old age.^17^, ^18^ Loss of RECQL5 also results in hypersensitivity to camptothecin (CPT), an inhibitor of topoisomerase I (Top I), but not to other DSB‐causing agents, in mouse cells and human cancer cells,^19^, ^20^ suggesting that RECQL5 plays an important role in dealing with replication stress. Consistent with the sensitivity to CPT caused by the loss of RECQL5, it has been proposed that RECQL5 helps replication fork reversal, probably through promoting the formation of double helix between two newly synthesized strands at a stalled replication fork.^16^, ^19^, ^21^, ^22^

Here, we report that RECQL5 is required for the maintenance of genome stability of TNBC cells. Compromising its function in TNBC cells results in persistence of DNA damage, G2 arrest, and ultimately, cessation of proliferation. Together with the observation that RECQL5 is dispensable in normal cells, our results suggest RECQL5 as a potential TNBC‐specific therapeutic target.

## 2 MATERIALS AND METHODS

### 2.1 Reagents

Dulbecco's modified Eagle's medium (DMEM), RPMI‐1640 Medium, fetal bovine serum (FBS), and antibiotics were purchased from Gibco (Grand Island, New York). The antibodies used in this study were as follows: RECQL5 (A302‐520A, 1:2000 WB, Bethyl Lab, Montgomery, TX); γH2AX (05‐636, 1:500 IF, Millipore, Billerica, MA); γH2AX (A300‐081A, 1:500 IF, Bethyl Lab); 53BP1 (NB100‐304, 1:500 IF, Novus Biologicals, Littleton, CO); cyclin A (SC‐271682, 1:50 IF, Santa Cruz Biotechnology, Santa Cruz, CA); BRCA1 (SC‐6954, 1:50 IF, Santa Cruz Biotechnology); BrdU (347580, 1:40 IF, BD Biosciences, San Jose, CA); EdU Apollo488 Kit (C10310‐3, RIB‐BIO, Guangzhou, China). Phospho‐Chk1‐Ser317 (12302, 1:1000 WB, Cell Signaling, Beverly, MA), Phospho‐Chk1‐Ser345 (2348, 1:1000 WB, Cell Signaling); CHK1 (ab32531, 1:1000 WB, Abcam, Cambridge, UK); GAPDH (60004‐1‐1g, 1:5000 WB, Proteintech, Wuhan, China); Tubulin (66240‐1‐1g, 1:5000 WB, Proteintech). Secondary antibodies conjugated to horseradish peroxidase were purchased from Santa Cruz Biotechnology. Secondary antibodies for immunofluorescence staining were anti‐mouse, ‐goat or ‐rabbit Alexa fluor 488 or 594 from Jackson ImmunoResearch Laboratories (West Grove, Pennsylvania). siRNAs were synthesized by GenePharma (Suzhou, China). Chemicals were obtained from Sigma (St. Louis).

shRNAs were constructed in pLKO.1 with following sequences: negative control, 5’‐TTCTCCGAACGTGTCACGT‐3’; shRECQL5‐1, 5’‐TTGTCGCCCATTGGAATATTG‐3’; shRECQL5‐2, 5’‐GTACGCTGAAGAAGGTCTTTG‐3’. For *RECQL5* expression, the cDNA (wild type or silent mutated to resist si/shRNA) was cloned into lentiviral vector pHAGE.

### 2.2 Cell culture and transfection

MDA‐MB 231, MDA‐MB 436, MDA‐MB 157, MDA‐MB 468, HCC1806, HS578T, BT549, SUM159, and T47D cell lines were purchased from American Type Culture Collection (ATCC). HCC1937, MCF7, and ZR75‐1 cells were obtained from The Cell Bank of Type Culture Collection of Chinese Academy of Sciences (Beijing, China). The above TNBC or non‐TNBC cells were cultured in DMEM, RPMI‐1640 or F12 Medium with 10% FBS in a humidified atmosphere containing 5% CO_2_ at 37°C. Plasmids used in the work were generated through standard cloning methods. Lentiviruses‐carrying overexpression or knockdown elements were produced in the lab and used to infect the above cell lines with MOI (multiplicity of infection) ＞1. The infected cells were selected with puromycin treatment (4 μg/mL for 2 days).

### 2.3 Assays for cell proliferation

For MTS assay, after lentiviral infection and selection, the cells were trypsinized and reseeded in 96‐well plates at a density of 3000 cells/well and cultured for the indicated times. At the end of incubation, proliferation was analyzed using a colorimetric assay (MTS, Promega, Madison, WI). Briefly, 20 μL MTS was added to 100 μL fresh complete culture medium in each well, and the cells were incubated for 2 hours before the absorbance of the formazan product at 490 nm was measured.

To detect the effects of replication inhibitors on non‐TNBC cell viability, T47D cells were seeded at 5000 cells/well in 96‐well plates and then treated with virous concentrations of CPT (0, 2.5, 5, 10 nmol/L) or 5‐Fluorouracil (5‐FU) (0, 25, 50, 100, 200 μg/mL) for 48 hours. Following incubation, MTS assays were performed.

Cellular senescence was assessed by measuring senescence‐associated β‐galactosidase activity as described before.^23^

### 2.4 Western blotting analysis

The cells were lysed with RIPA lysis buffer (Applygen Technologies Inc, Beijing, China) supplemented with protease inhibitor cocktail (Roche Diagnostics, Mannheim, Germany). Equal amounts of proteins were loaded to and separated in a SDS‐polyacrylamide gel, and transferred to a polyvinylidene difluoride membrane (PVDF, Merck Millipore, Massachusetts). The membrane was incubated for 1 hours in blocking buffer (5% nonfat dry milk in TBST) and with primary antibodies at 4°C overnight. After three washes with TBST, the membrane was incubated for 1 hours at room temperature with horseradish peroxidase (HRP)‐conjugated secondary antibodies. The membrane was then washed three times and visualized with SuperSignal™ West Pico Chemiluminescent Substrate (Thermo Fisher Scientific, San Jose, CA). Expression of GAPDH or Tubulin was routinely used as a loading control.

### 2.5 Immunostaining

Cells after indicated treatment were plated on coverslips, fixed with 4% paraformaldehyde for 15 minutes, permeabilized in PBS containing 0.5% Triton X‐100 for 5 minutes, and blocked with 5% BSA in PBS for 1 hours at room temperature, followed by incubation with primary antibodies at 4°C overnight. After three washes in PBS, the coverslips were incubated with secondary antibodies for 20 minutes at 37°C. All images were taken on a Nikon Ni‐E microscope (Nikon Corporation, Tokyo, Japan), with identical exposure times for each sample.

### 2.6 Replication restart and indirect fork reversal assay

Replication restart assay was performed as described previously.^22^ The fork reversal assay was based on BrdU staining as previously described.^24^ MDA‐MB 231 cells infected with shRNAs were seeded on coverslips. Cells were pulsed with 10 μmol/L BrdU for 15 minutes, washed three times in fresh media and treated with 1 μmol/L CPT (Sigma, St. Louis) for 160 minutes or 3 mmol/L hydroxyurea (HU) (Sigma) for 6 hours immediately. Cells were preextracted with 0.5% Triton X‐100, fixed with 4% formaldehyde and immunostained with anti‐BrdU antibody under native conditions. Images were captured with a Nikon Ni‐E microscope and analyzed with Columbus.

### 2.7 Fluorescence activated cell sorting

The cells were trypsinized and washed once with cold PBS. For cell cycle analysis, the cells were fixed in 70% ice‐cold EtOH, spun down, washed with cold PBS, and incubated in PBS containing propidium iodide (PI, 50 μg/mL) and RNase A (50 μg/mL) for 30 minutes at room temperature. The PI‐stained single cell suspension was analyzed on a BD LSRFortessa SORP Flow Cytometer (BD Biosciences). ModFit LT software (Verity Software House, Topsham, ME) was used to analyze the DNA patterns and cell cycle stages.

### 2.8 Tumor xenograft

Luciferase‐expressing HCC1806 cells were stably infected with lentiviruses encoding RECQL5 or control shRNAs and used to inoculate BALB/c nude mice purchased at approximately 3‐4 week of age from Charles River Laboratories. For each group, at least eight mice were used. All animals were kept in an environmentally controlled facility and given free access to water and a standard diet. All animal experiments were performed according to the guidelines approved by the Animal Care and Use Committee of National Center for Protein Sciences at Beijing. For tumor growth evaluation, 1 × 10^6^ RECQL5‐shRNA treated or control HCC1806 cells were injected into the left inguinal mammary fat pads. Tumor xenografts in each group were monitored every 5 days with an in vivo imaging system (IVIS, PerkinElmer). Two weeks after the inoculation, the tumors were removed, and the tumor volume was measured.

### 2.9 RECQL5 expression analysis and KM‐Plotter Survival analysis

The website UALCAN (http://ualcan.path.uab.edu/index.html) was used to analyze the RECQL5 mRNA expression based on the TCGA breast RNA‐Seq data set. The Kaplan‐Meier Plotter software (http://kmplot.com/analysis) was used to analyze the relevance of RECQL5 mRNA expression (Affymetrix (Santa Clara, CA) ProbeID 34063_at) to the overall survival (OS) in the 255 TNBC patients.

## 3 RESULTS

### 3.1 TNBC cells display high levels of endogenous DNA damage

**Figure 1:**
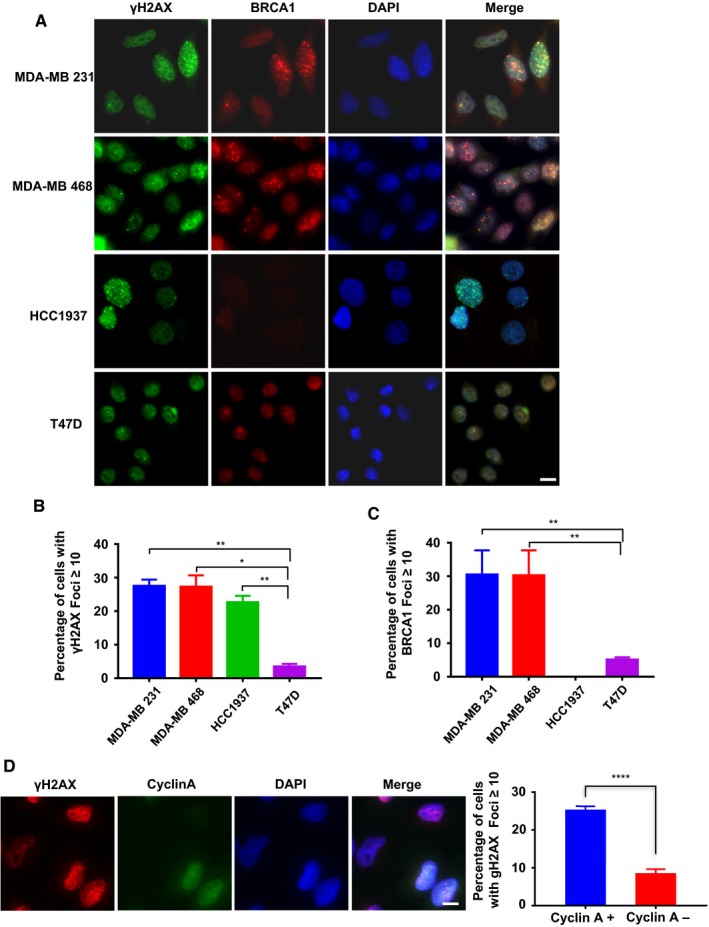
DNA damage in TNBC cells. A, Immunofluorescence (IF) staining of DNA damage marker γH2AX and BRCA1 in TNBC cell lines, MDA‐MB 231, MDA‐MB 468, and HCC1937 and non‐TNBC cell line T47D. Scale bars, 10 μm. B and C, Quantitation of results in A, At least 100 cells were analyzed for each cell line. Results are mean ± SEM D, MDA‐MB 231 cells were co‐immunostained with antibodies against γH2AX (Red) and cyclin A (Green), Scale bars, 10 μm. Quantitation of results in D, the γH2AX foci in both cyclin A‐positive and cyclin A‐negative nuclei were counted. Results are mean ± SEM (n ≥ 100)

We first examined γH2AX focus formation in two *BRCA1* wild type TNBC cell lines, MDA‐MB 231 and MDA‐MD 468 and one *BRCA1‐*mutant TNBC cell line HCC1937 (homozygous for the *BRCA1 *5382insC mutation). As shown in Figure 1A,1, about 25% of the cells from these three lines contained more than 10 γH2AX foci, whereas only few of non‐TNBC cells, T47D, stained positive. Staining for BRCA1 foci yielded similar results except in HCC1937 which lacks *BRCA1* and hence serviced as a negative control. (Figure 1A,C). Further, γH2AX foci were readily detectable in additional TNBC cell lines (Figure S1A,B), as well as another marker, 53BP1 (Figure S1C,D). Staining in two other non‐TNBC cell lines MCF7 and ZR75‐1 showed no sign of DNA damage (Figure S1A,B) (with ≥5 foci/cell, an even lower standard than that in Figure 1B, for this DNA damage marker). These results are consistent with the result from gene expression profiling experiment demonstrating an enrichment of DNA damage response genes in TNBC cells.^25^

The presence of elevated levels of both γH2AX and BRCA1 foci in TNBC cells suggests that the DNA damages are in the form of double‐strand breaks (DSBs) and are undergoing HR‐mediated repair. Co‐staining γH2AX with cyclin A showed that more than 25% of cyclin A‐positive cells (those in S/G2) were γH2AX‐positive (Figure 1D), whereas <10% of cyclin A‐negative cells were, indicating that DNA damage in TNBC cells is generated in S phase, most likely a result of replication fork collapse.^26^ This result also indicates that unlike non‐TNBC cell lines, a high proportion of the TNBC cells in S phase experience replication stress. The damages detected in cyclin A‐negative cells were probably from previous S phase that did not get repaired.

### 3.2 Disruption of RECQL5 function enhances DNA damage in TNBC cells

**Figure 2:**
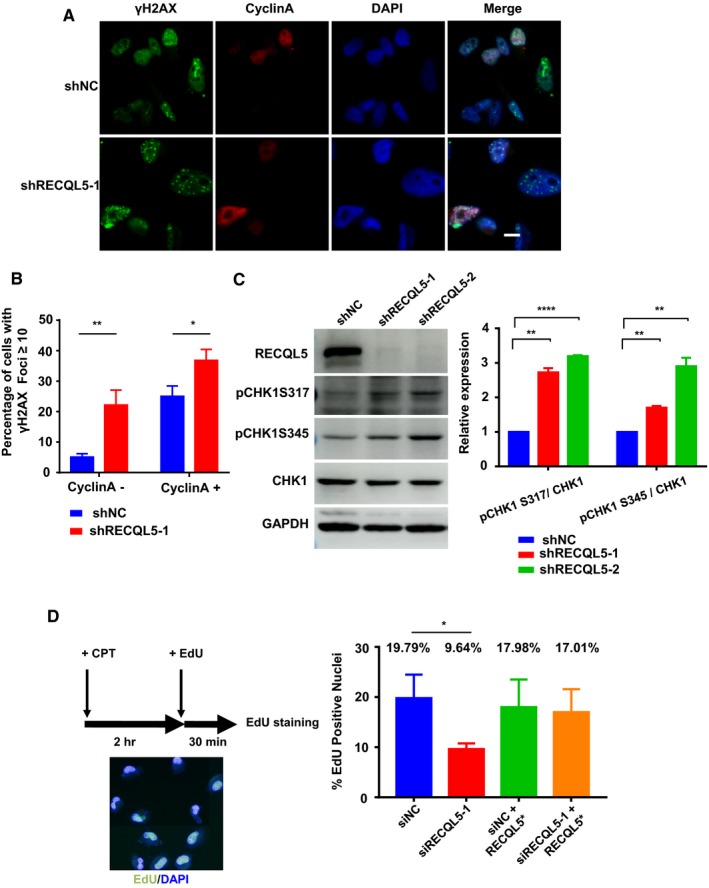
RECQL5 depletion enhances DNA damage in TNBC cells. A, MDA‐MB 231 cells with or without RECQL5 depletion were co‐immunostained with antibodies against γH2AX (Green) and cyclin A (Red), Scale bars, 10 μm. B, Quantitation of results in A, the γH2AX foci in both cyclin A‐positive and cyclin A‐negative nuclei were counted. Results are mean ± SEM (n ≥ 100). *, *P* < 0.05, **, *P* < 0.01 (Student's *t* test). C, Western blotting analysis of CHK1 phosphorylation (pCHK1 S317 and pCHK1 S345) in MDA‐MB 231 cells with or without RECQL5 depletion. D, Replication restart assay. RECQL5* indicates the expression of a siRNA‐resistant version of RECQL5. Results are mean ± SEM (n ≥ 100), *, *P* < 0.05 (Student's *t* test)

RECQL5 was proposed to play a role in maintaining replication fork stability through promoting replication fork reversal and helping restart stalled replication forks.^16^, ^19^, ^21^ Compromising its function can result in sensitivity to replication stress inducers.^19^, ^22^ We therefore asked if RECQL5 played any roles in TNBC cells where there seems to be increased levels of replication stress. The helicase was depleted in MDA‐MB 231 cells via shRNA and γH2AX focus formation was assessed in these and control cells. As shown in Figure 2A,B, it is clear that loss of RECQL5 function increased γH2AX focus formation. Interestingly, the increase was unproportional between cyclin A‐positive subpopulation and cyclin A‐negative subpopulation. Much more cyclin A‐negative cells than cyclin A‐positive cells now became γH2AX‐positive (Figure 2B), which most likely resulted from cells leaving S phase without replication‐related damage repaired due to lack of RECQL5 function. Consistent with that, we observed an increase in the level of CHK1 phosphorylation, suggesting the activation of replication checkpoint upon depletion of RECQL5 (Figure 2C). We next examined the effect of RECQL5 depletion in MDA‐MB 436, a *BRCA1*‐mutant TNBC cell line. Again, as shown in Figure S2A, RECQL5 depletion increased the number of γH2AX foci dramatically. However, depleting RECQL5 in T47D, a non‐TNBC breast cancer cell line did not cause any increases in the level of endogenous DNA damage (Figure S2B), suggesting that RECQL5 is not as irreplaceable in non‐TNBC cells as in TNBC cells.

To confirm the function of RECQL5 in dealing with replication stress in TNBC cells, we induced high levels of replication stress in control and RECQL5‐depleted MDA‐MB 231 cells by treating them with HU or CPT and then looked for replication restart or the formation of single strand DNA (ssDNA) which is a measurement of replication fork reversal.^24^, ^27^ As shown in Figure 2D, CPT treatment blocked replication restart greatly in RECQL5 knockdown cells, but such a blockage could be relieved with reexpression of a siRNA‐resistant version of RECQL5 (Figure 2D and S3A). ssDNA formation was also reduced in both HU and CPT‐treated RECQL5‐depleted cells (Figure S3B,C). Given the heterogeneity of knockdown effect, we were not surprised to find that there were not only reduced number of cells stained positive ssDNA (Figure S3B,C), but also that the intensity of BrdU staining in the remaining BrdU‐positive cells was reduced as well (Figure S3C).

Having established the role of RECQL5 in combating replication stress in TNBC cells, we wondered if the helicase also is required in non‐TNBC breast cancer cells when they were challenged with replication stress. To that end, we treated T47D, an ER‐positive breast cancer cell line, with either CPT or 5‐FU to induce replication stress and measured the ability of the control and RECQL5‐depleted cells to survive. As shown in Figure S4A,B, RECQL5‐depletion severely impaired the survivability of the cells treated with CPT or 5‐FU. This result is consistent with previous reports that the loss of RECQL5 function sensitizes cells to replication stress.^19^, ^20^

### 3.3 *RECQL5 is essential for the growth of TNBC cells *in vitro

**Figure 3:**
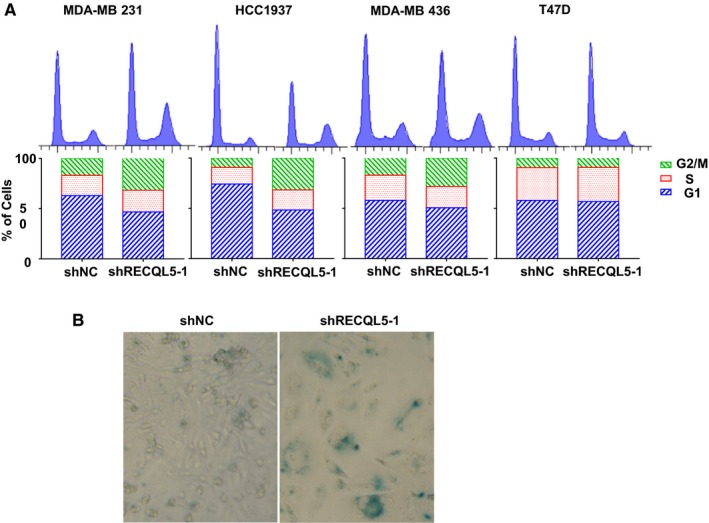
RECQL5 depletion disrupts cell cycle progression and cell viability. A, Cell cycle analysis of MDA‐MB 231, HCC1937, MDA‐MB 436 and T47D cells with or without RECQL5 depletion. B, Senescence‐associated β‐galactosidase staining of MDA‐MB 231 cells with or without RECQL5 depletion

The results so far indicate that RECQL5 plays a critical role in maintaining genome integrity in TNBC cells by helping relieve replication stress. We next asked what is the effect of RECQL5 depletion on cell proliferation. First, we analyzed cell cycle distribution in control and RECQL5‐depleted MDA‐MB 231 and T47D cells. Consistent with the increase in DNA damage levels and CHK1 activation (Figure 2), we found that RECQL5 depletion led to G2 arrest in MDA‐MB 231 cells, but had little effect on non‐TNBC cells T47D (Figure 3A). We then tested HCC1937 and MDA‐MB 436. Again, RECQL5*‐*depleted HCC1937 and MDA‐MB 436 entered G2 arrest (Figure 3A). Over time, RECQL5‐depleted MDA‐MB 231 cells stopped proliferation altogether, displaying a large flattened morphology, suggesting senescence. We therefore stained for senescence‐associated β‐galactosidase activity. Indeed, the cells were positive for the senescence marker (Figure 3B). Interestingly, there are also cells in the control stained positive (Figure 3B), which is not unexpected since we could see a fraction of cyclin A‐negative cells with persistent DNA damage in undisturbed cell population already (Figure 2A,B). These cells likely incurred too much damage in previous S phase which did not fully repair. The DNA damage checkpoint then prevented them from entering mitosis and perhaps pushed them into senescence as a consequence.

**Figure 4:**
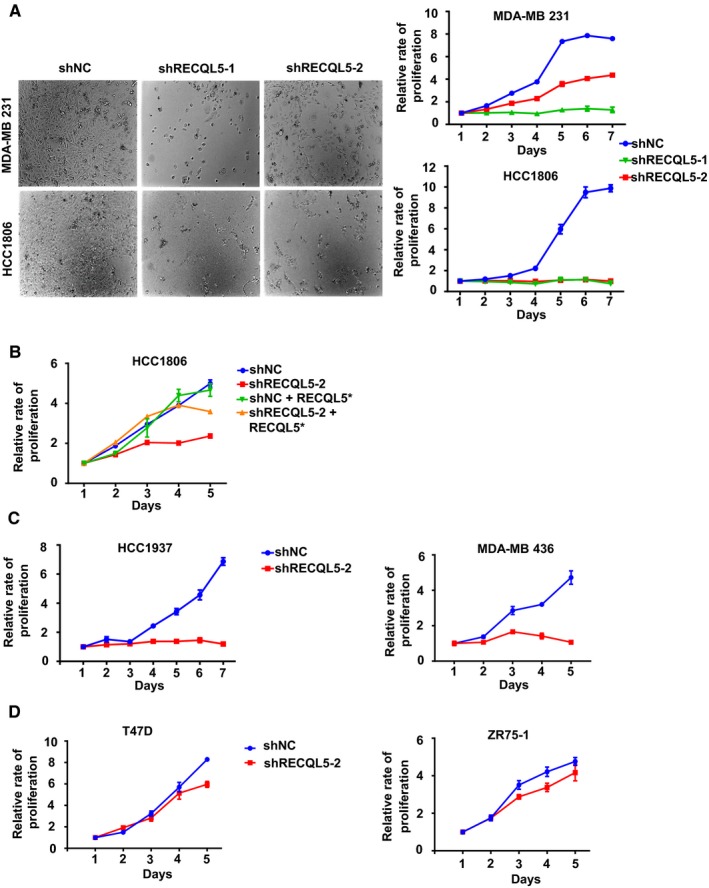
RECQL5 is required for the growth of TNBC cells. A, Micrographs and Growth curves of MDA‐MB 231 and HCC1806 cells grown in 96‐well plate for 7 days. B, Growth curves of HCC1806 cells. RECQL5* indicates the expression of a shRNA‐resistant version of RECQL5. C, Growth curves of HCC1937 and MDA‐MB 436 cells. D, Growth curves of non‐TNBC cell lines T47D and ZR75‐1

Next, we examined the effect of RECQL5 depletion on the proliferation of other TNBC cell lines. Like MDA‐MB 231, HCC1806 is another *BRCA1* wild type TNBC cell line which also failed to proliferate when *RECQL5* expression was silenced with two different shRNAs (Figure 4A and S5A). The effect of the second shRNA construct (shRECQL5‐2) (Figure 4B) could be rescued by reexpression of a resistant version of RECQL5 (Figure S5B). Moreover, HCC1937 and MDA‐MB 436, both lacking *BRCA1,* were examined for their ability to proliferate upon RECQL5 depletion. As expected, these two lines also failed to proliferate (Figure 4C anD S5A). We further tested the essentiality of *RECQL5* in two ER‐positive cell lines (T47D and ZR75‐1). As shown in Figure 4D and S5A, the depletion of RECQL5 had little effect on the growth of T47D and ZR75‐1. These data indicate that RECQL5 is specifically required in TNBC cells.

### 3.4 RECQL5 is required for xenograft growth of TNBC cells

**Figure 5:**
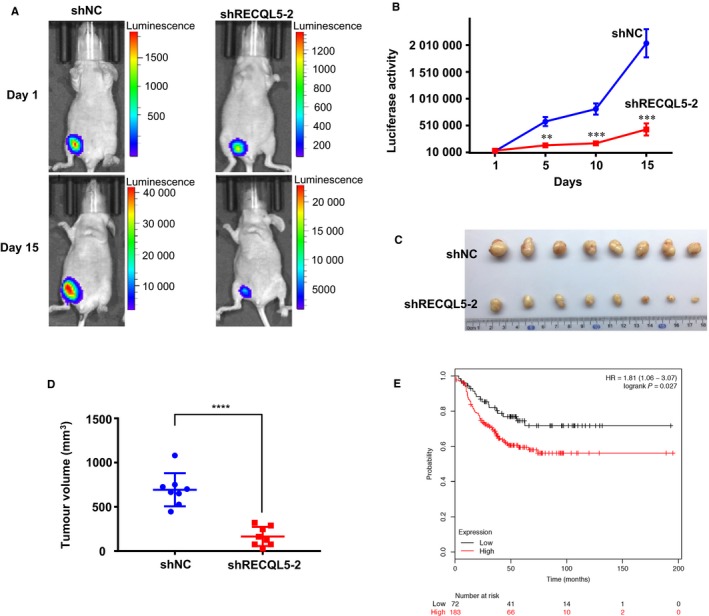
RECQL5 depletion impedes xenograft growth of TNBC cells. Luciferase‐expressing HCC1806 cells with or without RECQL5 depletion were injected into the bottom left mammary glands of BALB/c nude female mice (1 × 10^6^ cells per mouse, eight mice per group). A, Bioluminescent images. B, Growth curve analysis of tumor xenografts. C, Photographs of the tumors. D, Tumor volumes at day 15. Data are mean ± SEM (B, D). **, *P* < 0.01; ***, *P* < 0.001; ****, *P* < 0.0001 (Student's *t* test). E, KM Plotting of TNBC patients. Probe used was 34063_at (Affy ID), expression range 13‐302, and cutoff value was 71. Plots showed overall survival in TNBC patients with low (black trace) or high (red trace) levels of RECQL5 expression. HR, hazard ratio

Having established that RECQL5 is required for the growth of TNBC cells in vitro, we decided to determine if RECQL5 was also required for in vivo growth of TNBC cells. Luciferase‐expressing HCC1806 cells were infected with lentiviruses carrying control or RECQL5 shRNA, selected with puromycin, and injected orthotopically into the left inguinal mammary fat pads of female nude mice. Tumor growth was monitored by bioluminescent imaging every 5 days. As shown in Figure 5A,B, RECQL5‐depleted xenografts grew much slower than controls. At the end of experiment (day 15), control tumors had grown to much larger sizes than that of RECQL5*‐*depleted ones (Figure 5C,D).

Through analyzing the Cancer Genome Atlas (TCGA) breast cancer RNA‐Seq data sets,^28^ we found that RECQL5 mRNA levels were modestly elevated in two breast tumor subtypes, luminal and triple‐negative compared with normal breast tissue (Figure S5C). Consistent with the data, RECQL5 protein was upregulated in many luminal and TNBC cell lines relative to normal breast cell line MCF10A (Figure S5D). Next, we asked whether the expression levels of RECQL5 correlated with prognosis of TNBC patients. We reasoned that high levels of RECQL5 expression would enable TNBC cells to deal with replication stress better and therefore better chances to survive and proliferate than those cells with lower levels of expression. Using KM‐Plotter,^29^ we analyzed publicly available breast cancer data. Indeed, elevated RECQL5 expression levels predicted a less favorable overall survival in patients with TNBC (255 cases, *P* = 0.027, Figure 5E and Table. S1).

## 4 DISCUSSION

Among various types of breast cancers, TNBC is unique, not only in its hormone receptor status but also in its association with high levels of endogenous DNA damage manifested by a gene expression profile enriched with DNA damage response genes^6^ and by histological observation in clinical samples.^30^ We showed here that in all TNBC cell lines examined there were increased levels of γH2AX. These cells also show BRCA1 foci, which, together with the finding that the damage was mostly detected in S/G2 phase cells, suggesting that the damage stems from problems in DNA replication, most likely from replication fork collapse. Replication stress arises from a number of complications with the chromatin including fragile sites, mis‐coordination between nucleotide synthesis and replication, oncogene activation, single strand lesions, etc.^31^ It is unclear what drives the high levels of replication stress in TNBC cells, but single strand lesions derived from oxidative damage are the likely culprit. Indeed, in addition to genomic DNA, other cellular components are also under oxidative attack in TNBCs as detected by oxidative profiling (lipid peroxidation and nitric oxide) of triple‐negative breast tumors.^32^ Cellular redox state is carefully regulated by a large number of proteins including BRCA1.^33^, ^34^ It is possible that genetic defects in redox regulators including *BRCA1* may be behind the replication stress in TNBC. Furthermore, mutations in DNA damage response and repair genes other than *BRCAs* 30, 35 may also contribute to the generation of replication stress in TNBC.

A stalled replication fork can be stabilized via fork reversal or may collapse and form one‐ended double‐strand break that requires HR‐mediated repair to fix. If left unfixed, collapsed replication forks could lead to broken chromosomes and subsequent gross chromosomal instability. Our data suggest that TNBC cells are under replication stress and constantly generating double‐strand breaks as demonstrated by γH2AX staining. It should be pointed out that there is heterogeneity in terms of the degree of replication stress experienced by individual cells. Those under highest level of stress would not be able to survive. They might die off or enter senescence. Indeed, we could see senescent cells already present in undisturbed cultures of MDA‐MB 231 cells (Figure 3B). Thus, as a population, TNBC cells, even those without *BRCA1* function (such as HCC1937), are viable and still proliferating, despite some individual cells are constantly leaving the population. However, when the function of RECQL5 is compromised, the severity of replication stress increases so much that more and more cells die off or senesce, and ultimately, proliferation ceases. For *BRCA1* mutant cells, compromising RECQL5 would be equivalent to treating them with PARP inhibitors.

Polymorphisms in *RECQL5* were found to be associated with increased susceptibility to breast cancer.^36^ Together with the tumor phenotype in the deficient mice,^37^ this observation suggests that *RECQL5* is a tumor suppressor. Given the function of RECQL5 in DNA metabolism, it is likely that compromising its function could lead to genome instability and consequently tumorigenesis. On the other hand, a large scale expression profiling of *RECQL5* in human breast cancer showed that high expression of the helicase is often associated with bad tumor grades and poor prognosis.^38^ In line with that, our analysis of RECQL5 expression data also suggests that higher levels of expression are correlated with poorer prognosis of TNBC patients (Figure 5E). These observations suggest that *RECQL5* could also function as an oncogene. Although neither the tumor suppressor function nor the oncogene function of *RECQL5* is particularly strong.

The high levels of endogenous DNA damage in TNBC cells make *RECQL5* essential in TNBCs, and therefore a potential drug target against TNBC. It is highly likely that other RecQ family members are essential as well. However, these other members seem more important than *RECQL5* as their deficiencies cause severe problems such as premature aging. Thus, targeting RECQL5 would be a better choice.

## CONFLICTS OF INTEREST

Authors declare no conflicts of interest for this article.

## Data Availability

The data that support the findings of this study are available from the corresponding author upon reasonable request.

## References

[1] Negrini S , Gorgoulis VG , Halazonetis TD . Genomic instability–an evolving hallmark of cancer. Nat Rev Mol Cell Biol. 2010;11:220‐228. 20177397 10.1038/nrm2858

[2] Chen CC , Feng W , Lim PX , Kass EM , Jasin M . Homology‐directed repair and the role of BRCA1, BRCA2, and related proteins in genome integrity and cancer. Ann Rev Cancer Biol. 2018;2:313‐336. 30345412 10.1146/annurev-cancerbio-030617-050502 PMC6193498

[3] de Ruijter TC , Veeck J , de Hoon JP , van Engeland M , Tjan‐Heijnen VC . Characteristics of triple‐negative breast cancer. J Cancer Res Clin Oncol. 2011;137:183‐192. 21069385 10.1007/s00432-010-0957-x PMC3018596

[4] Rakha EA , Ellis IO . Triple‐negative/basal‐like breast cancer: review. Pathology. 2009;41:40‐47. 19089739 10.1080/00313020802563510

[5] Reis‐Filho JS , Tutt AN . Triple negative tumours: a critical review. Histopathology. 2008;52:108‐118. 18171422 10.1111/j.1365-2559.2007.02889.x

[6] Lehmann BD , Bauer JA , Chen XI , et al. Identification of human triple‐negative breast cancer subtypes and preclinical models for selection of targeted therapies. J Clin Investig. 2011;121:2750‐2767. 21633166 10.1172/JCI45014 PMC3127435

[7] Lakhani SR , Van De Vijver MJ , Jacquemier J , et al. The pathology of familial breast cancer: predictive value of immunohistochemical markers estrogen receptor, progesterone receptor, HER‐2, and p53 in patients with mutations in BRCA1 and BRCA2. J Clin Oncol. 2002;20:2310‐2318. 11981002 10.1200/JCO.2002.09.023

[8] Turner N , Tutt A , Ashworth A . Hallmarks of 'BRCAness' in sporadic cancers. Nat Rev Cancer. 2004;4:814‐819. 15510162 10.1038/nrc1457

[9] Shiu KK , Tan DS , Reis‐Filho JS . Development of therapeutic approaches to 'triple negative' phenotype breast cancer. Expert Opin Therap Targets. 2008;12:1123‐1137. 18694379 10.1517/14728222.12.9.1123

[10] Croteau DL , Popuri V , Opresko PL , Bohr VA . Human RecQ helicases in DNA repair, recombination, and replication. Annu Rev Biochem. 2014;83:519‐552. 24606147 10.1146/annurev-biochem-060713-035428 PMC4586249

[11] Larsen NB , Hickson ID . RecQ Helicases: conserved guardians of genomic integrity. Adv Exp Med Biol. 2013;767:161‐184. 23161011 10.1007/978-1-4614-5037-5_8

[12] Cybulski C , Carrot‐Zhang J , Kluźniak W , et al. Germline RECQL mutations are associated with breast cancer susceptibility. Nat Genet. 2015;47:643‐646. 25915596 10.1038/ng.3284

[13] Sun J , Wang Y , Xia Y , et al. Mutations in RECQL gene are associated with predisposition to breast cancer. PLoS Genet. 2015;11:e1005228. 25945795 10.1371/journal.pgen.1005228 PMC4422667

[14] Popuri V , Tadokoro T , Croteau DL , Bohr VA . Human RECQL5: guarding the crossroads of DNA replication and transcription and providing backup capability. Crit Rev Biochem Mol Biol. 2013;48:289‐299. 23627586 10.3109/10409238.2013.792770 PMC4563991

[15] Saponaro M , Kantidakis T , Mitter R , et al. RECQL5 controls transcript elongation and suppresses genome instability associated with transcription stress. Cell. 2014;157:1037‐1049. 24836610 10.1016/j.cell.2014.03.048 PMC4032574

[16] Islam MN , Fox D 3rd , Guo R , Enomoto T , Wang W . RecQL5 promotes genome stabilization through two parallel mechanisms–interacting with RNA polymerase II and acting as a helicase. Mol Cell Biol. 2010;30:2460‐2472. 20231364 10.1128/MCB.01583-09 PMC2863711

[17] Hu Y , Raynard S , Sehorn MG , et al. RECQL5/Recql5 helicase regulates homologous recombination and suppresses tumor formation via disruption of Rad51 presynaptic filaments. Genes Dev. 2007;21:3073‐3084. 18003859 10.1101/gad.1609107 PMC2081974

[18] Hu Y , Lu X , Barnes E , Yan M , Lou H , Luo G . Recql5 and Blm RecQ DNA helicases have nonredundant roles in suppressing crossovers. Mol Cell Biol. 2005;25:3431‐3442. 15831450 10.1128/MCB.25.9.3431-3442.2005 PMC1084310

[19] Hu Y , Lu X , Zhou G , Barnes EL , Luo G . Recql5 plays an important role in DNA replication and cell survival after camptothecin treatment. Mol Biol Cell. 2009;20:114‐123. 18987339 10.1091/mbc.E08-06-0565 PMC2613109

[20] Wang X , Lu X , Zhou G , Lou H , Luo G . RECQL5 is an important determinant for camptothecin tolerance in human colorectal cancer cells. Biosci Rep. 2011;31:363‐369. 21210765 10.1042/BSR20100108

[21] Blundred R , Myers K , Helleday T , Goldman AS , Bryant HE . Human RECQL5 overcomes thymidine‐induced replication stress. DNA Repair. 2010;9:964‐975. 20643585 10.1016/j.dnarep.2010.06.009

[22] Chen E , Ahn J , Sykes D , et al. RECQL5 Suppresses oncogenic JAK2‐induced replication stress and genomic instability. Cell Rep. 2015;13:2345‐2352. 26686625 10.1016/j.celrep.2015.11.037 PMC4691544

[23] Li M , Shin Y‐H , Hou L , et al. The adaptor protein of the anaphase promoting complex Cdh1 is essential in maintaining replicative lifespan and in learning and memory. Nat Cell Biol. 2008;10:1083‐1089. 19160489 10.1038/ncb1768 PMC2914158

[24] Fugger K , Mistrik M , Neelsen K , et al. FBH1 catalyzes regression of stalled replication forks. Cell Rep. 2015;10:1749‐1757. 25772361 10.1016/j.celrep.2015.02.028

[25] Ribeiro E , Ganzinelli M , Andreis D , et al. Triple negative breast cancers have a reduced expression of DNA repair genes. PLoS ONE. 2013;8:e66243. 23825533 10.1371/journal.pone.0066243 PMC3692506

[26] Allen C , Ashley AK , Hromas R , Nickoloff JA . More forks on the road to replication stress recovery. J Mol Cell Biol. 2011;3:4‐12. 21278446 10.1093/jmcb/mjq049 PMC3030971

[27] Couch FB , Bansbach CE , Driscoll R , et al. ATR phosphorylates SMARCAL1 to prevent replication fork collapse. Genes Dev. 2013;27:1610‐1623. 23873943 10.1101/gad.214080.113 PMC3731549

[28] Chandrashekar DS , Bashel B , Balasubramanya S , et al. UALCAN: a portal for facilitating tumor subgroup gene expression and survival analyses. Neoplasia. 2017;19:649‐658. 28732212 10.1016/j.neo.2017.05.002 PMC5516091

[29] Györffy B , Lanczky A , Eklund AC , et al. An online survival analysis tool to rapidly assess the effect of 22,277 genes on breast cancer prognosis using microarray data of 1,809 patients. Breast Cancer Res Treat. 2010;123:725‐731. 20020197 10.1007/s10549-009-0674-9

[30] Bartkova J , Tommiska J , Oplustilova L , et al. Aberrations of the MRE11‐RAD50‐NBS1 DNA damage sensor complex in human breast cancer: MRE11 as a candidate familial cancer‐predisposing gene. Mol Oncol. 2008;2:296‐316. 19383352 10.1016/j.molonc.2008.09.007 PMC5527773

[31] Zeman MK , Cimprich KA . Causes and consequences of replication stress. Nat Cell Biol. 2014;16:2‐9. 24366029 10.1038/ncb2897 PMC4354890

[32] Herrera A , Panis C , Victorino VJ , et al. Molecular subtype is determinant on inflammatory status and immunological profile from invasive breast cancer patients. Cancer Immunol Immunother. 2012;61:2193‐2201. 22618884 10.1007/s00262-012-1283-8 PMC11028631

[33] Saha T , Rih JK , Rosen EM . BRCA1 down‐regulates cellular levels of reactive oxygen species. FEBS Lett. 2009;583:1535‐1543. 19364506 10.1016/j.febslet.2009.04.005 PMC2744635

[34] Cao L , Xu X , Cao LL , et al. Absence of full‐length Brca1 sensitizes mice to oxidative stress and carcinogen‐induced tumorigenesis in the esophagus and forestomach. Carcinogenesis. 2007;28:1401‐1407. 17363841 10.1093/carcin/bgm060

[35] Tommiska J , Bartkova J , Heinonen M , et al. The DNA damage signalling kinase ATM is aberrantly reduced or lost in BRCA1/BRCA2‐deficient and ER/PR/ERBB2‐triple‐negative breast cancer. Oncogene. 2008;27:2501‐2506. 17982490 10.1038/sj.onc.1210885

[36] He YJ , Qiao ZY , Gao B , Zhang XH , Wen YY . Association between RECQL5 genetic polymorphisms and susceptibility to breast cancer. Tumour Biol. 2014;35:12201‐12204. 25394896 10.1007/s13277-014-2528-2

[37] Hosono Y , Abe T , Ishiai M , et al. Tumor suppressor RecQL5 controls recombination induced by DNA crosslinking agents. Biochem Biophys Acta. 2014;1843:1002‐1012. 24418621 10.1016/j.bbamcr.2014.01.005 PMC5525539

[38] Arora A , Abdel‐Fatah T , Agarwal D , et al. Clinicopathological and prognostic significance of RECQL5 helicase expression in breast cancers. Carcinogenesis. 2016;37:63‐71. 26586793 10.1093/carcin/bgv163 PMC4715235

