# Supplementary figures and images for: RECQL5 plays an essential role in maintaining genome stability and viability of triple‐negative breast cancer cells
Source: Cancer Med. 2019 Jun 23;8(10):4743–52. doi: 10.1002/cam4.2349 (PMC6712443; doi:10.1002/cam4.2349)

Figure S1

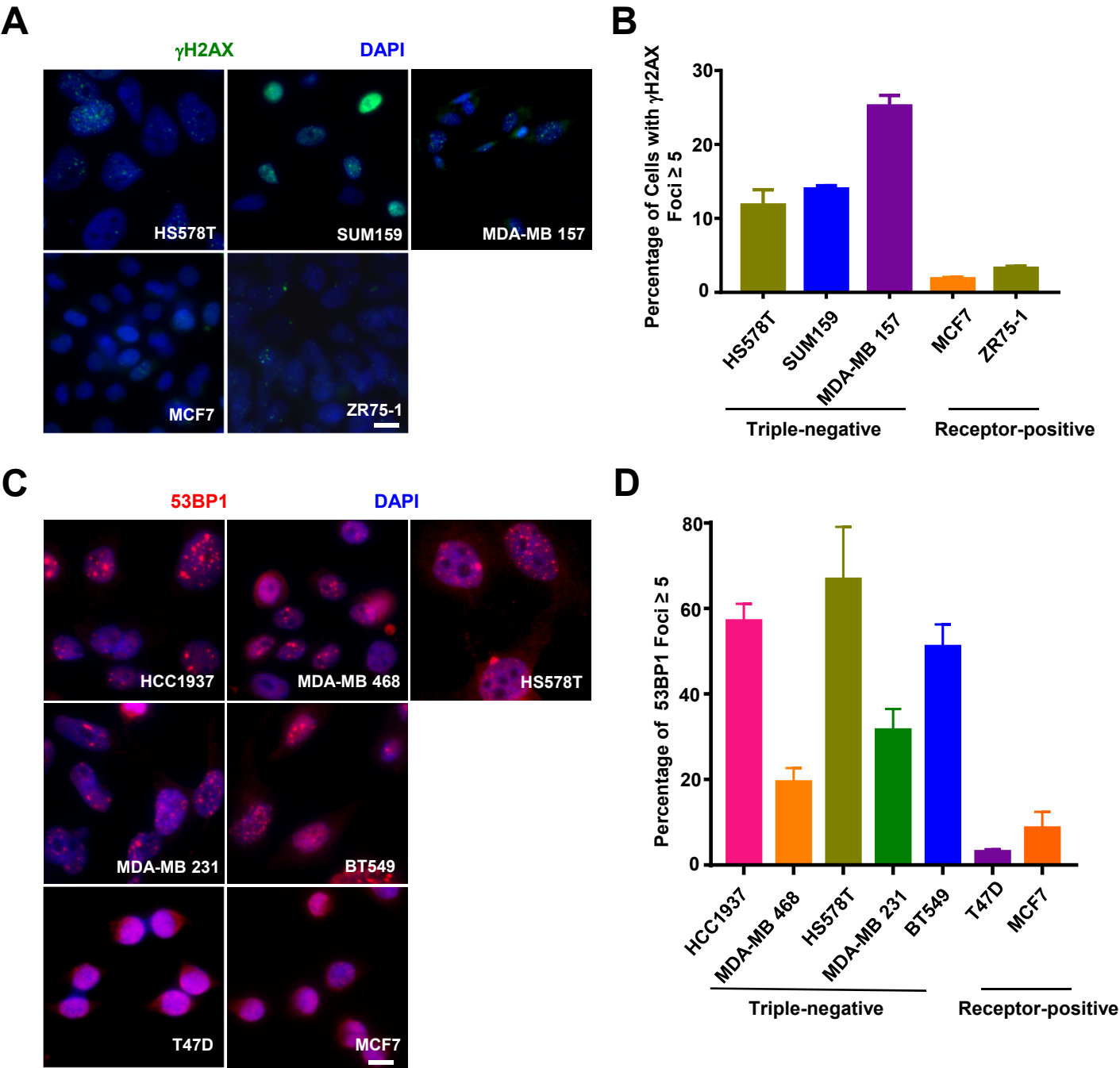

Supplement: Supplementary file 1 [file CAM4-8-4743-s001.pdf]

Figure S2

**A**

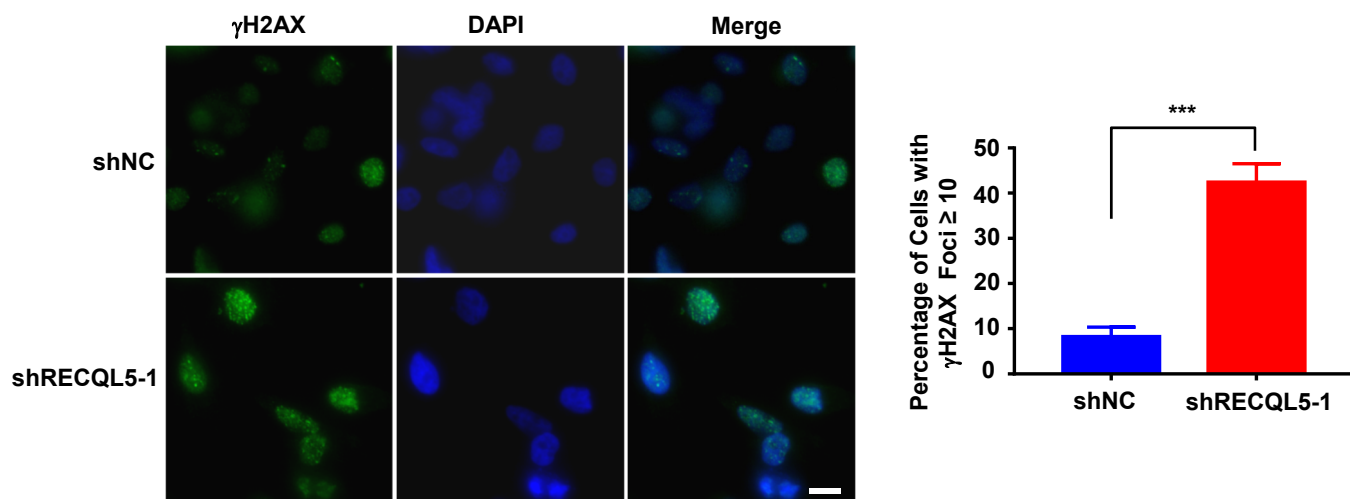

**B**

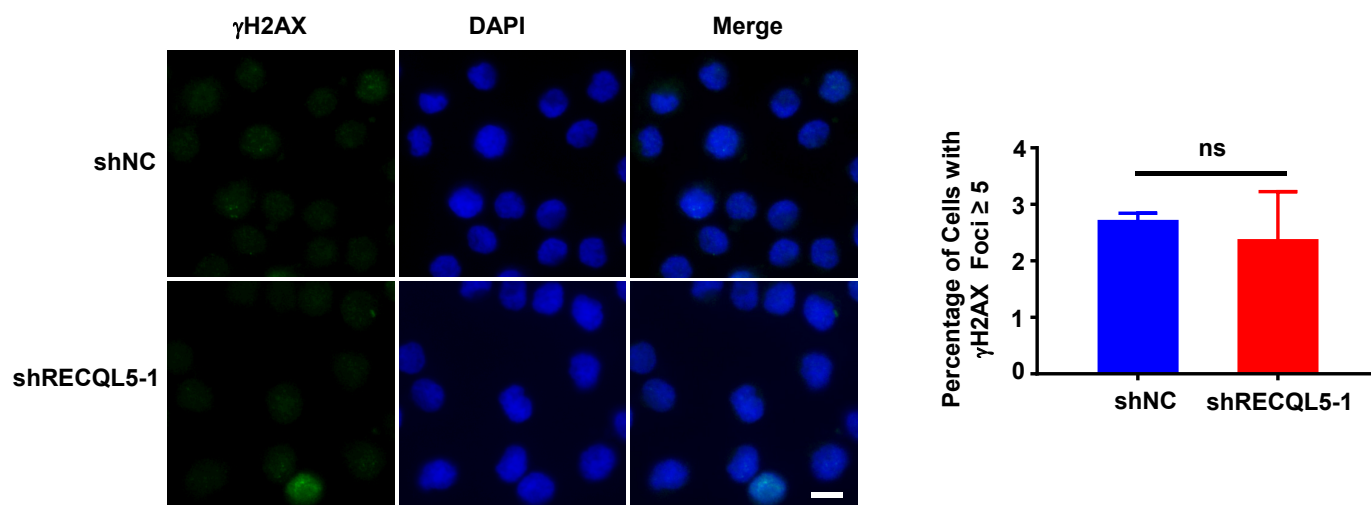

Supplement: Supplementary file 2 [file CAM4-8-4743-s002.pdf]

Figure S3

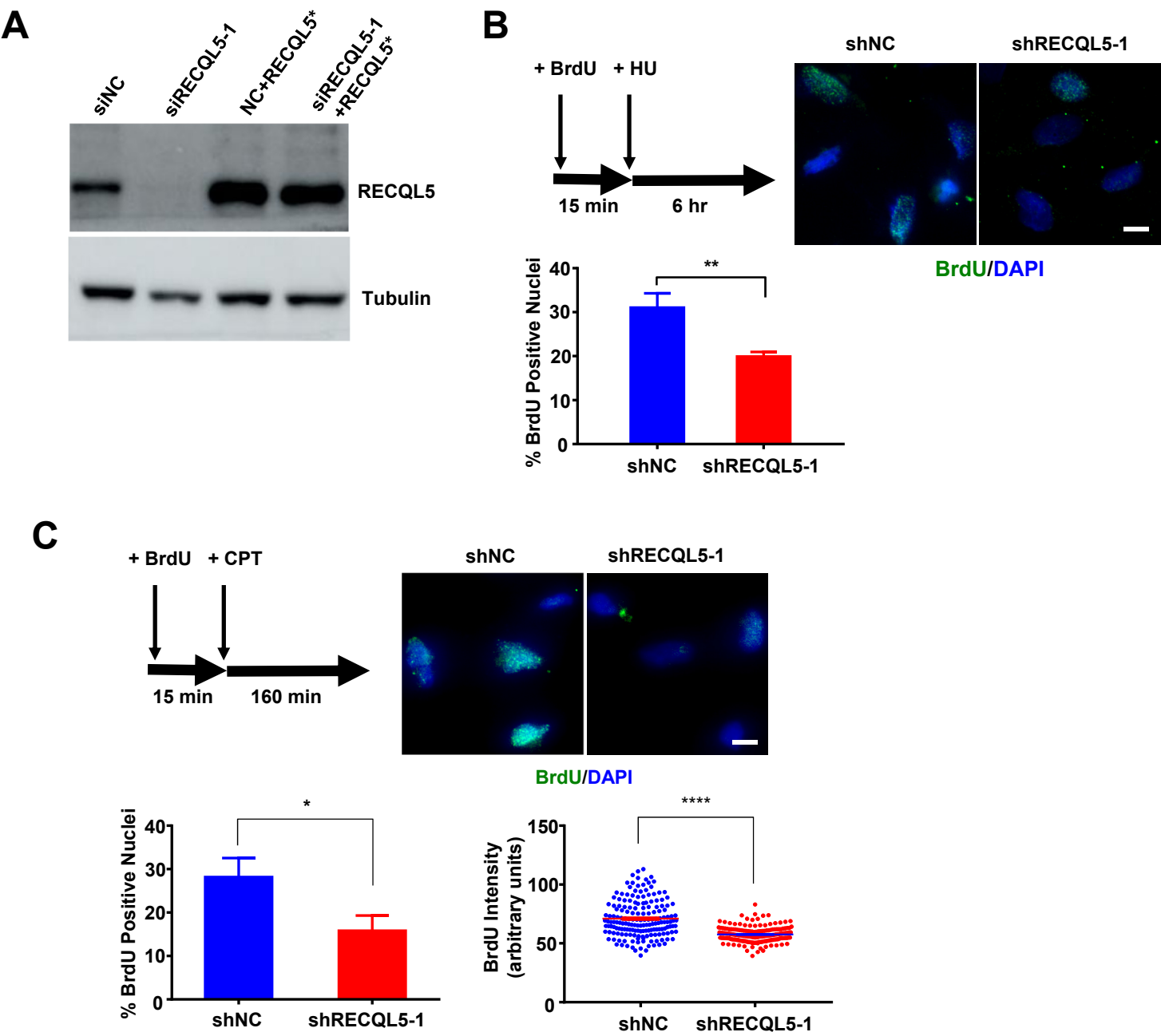

Supplement: Supplementary file 3 [file CAM4-8-4743-s003.pdf]

Figure S4

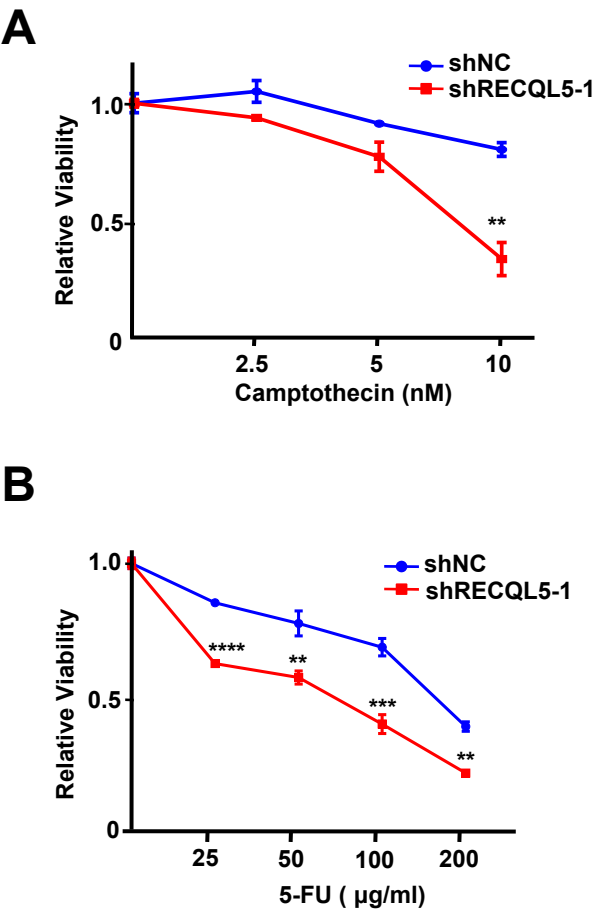

Supplement: Supplementary file 4 [file CAM4-8-4743-s004.pdf]

Figure S5

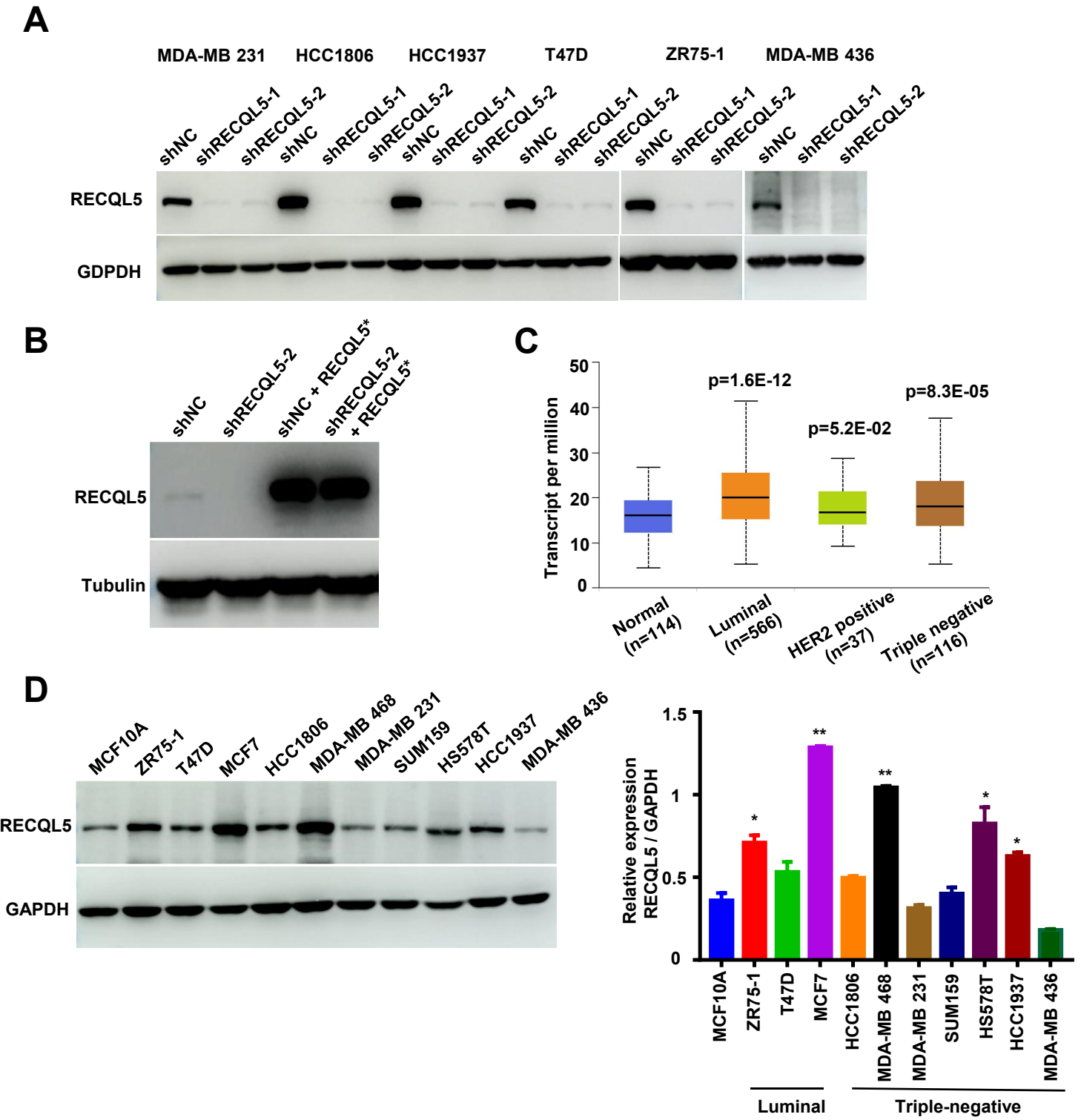

Supplement: Supplementary file 5 [file CAM4-8-4743-s005.pdf]
