# Supplementary material for: RECQL5 plays an essential role in maintaining genome stability and viability of triple‐negative breast cancer cells
Source: Cancer Med. 2019 Jun 23;8(10):4743–52. doi: 10.1002/cam4.2349 (PMC6712443; doi:10.1002/cam4.2349)
Supplement: Supplementary file 6 [file CAM4-8-4743-s006.docx]

| **Table. S1** | |  |  |
| --- | --- | --- | --- |
| Sample | Expression (1=high) | Time (months) | Event |
| 1 | 1 | 78 | 0 |
| 2 | 1 | 27 | 1 |
| 3 | 1 | 74 | 0 |
| 4 | 1 | 57 | 1 |
| 5 | 1 | 38 | 0 |
| 6 | 1 | 57 | 0 |
| 7 | 1 | 25 | 1 |
| 8 | 1 | 61 | 0 |
| 9 | 0 | 58 | 0 |
| 10 | 0 | 43 | 1 |
| 11 | 0 | 7 | 1 |
| 12 | 0 | 38 | 1 |
| 13 | 1 | 45 | 0 |
| 14 | 0 | 5 | 1 |
| 15 | 1 | 1 | 1 |
| 16 | 1 | 1 | 0 |
| 17 | 1 | 24 | 0 |
| 18 | 1 | 17 | 1 |
| 19 | 1 | 49 | 0 |
| 20 | 1 | 95 | 0 |
| 21 | 0 | 27 | 0 |
| 22 | 1 | 66.48 | 0 |
| 23 | 1 | 60.96 | 0 |
| 24 | 1 | 50.04 | 0 |
| 25 | 1 | 9 | 0 |
| 26 | 1 | 14.04 | 0 |
| 27 | 1 | 69.96 | 0 |
| 28 | 1 | 38 | 0 |
| 29 | 0 | 60 | 0 |
| 30 | 0 | 62 | 0 |
| 31 | 1 | 64 | 0 |
| 32 | 0 | 62 | 0 |
| 33 | 1 | 52 | 0 |
| 34 | 1 | 1 | 1 |
| 35 | 1 | 53 | 0 |
| 36 | 0 | 54 | 0 |
| 37 | 0 | 51 | 0 |
| 38 | 1 | 48 | 0 |
| 39 | 1 | 52 | 0 |
| 40 | 0 | 50 | 0 |
| 41 | 1 | 40 | 0 |
| 42 | 0 | 49 | 0 |
| 43 | 0 | 53 | 0 |
| 44 | 0 | 50 | 0 |
| 45 | 1 | 35 | 1 |
| 46 | 1 | 50 | 0 |
| 47 | 1 | 36 | 0 |
| 48 | 1 | 39 | 0 |
| 49 | 1 | 18 | 1 |
| 50 | 0 | 73 | 0 |
| 51 | 1 | 56 | 0 |
| 52 | 1 | 14 | 1 |
| 53 | 1 | 75 | 0 |
| 54 | 0 | 57 | 0 |
| 55 | 1 | 21 | 1 |
| 56 | 1 | 22.02739726 | 1 |
| 57 | 1 | 90.83835616 | 0 |
| 58 | 1 | 52.93150685 | 0 |
| 59 | 1 | 96.46027397 | 0 |
| 60 | 1 | 72.85479452 | 0 |
| 61 | 1 | 47.50684932 | 1 |
| 62 | 1 | 37.34794521 | 1 |
| 63 | 1 | 11.24383562 | 1 |
| 64 | 1 | 14.86027397 | 1 |
| 65 | 1 | 9.271232877 | 1 |
| 66 | 1 | 45.92876712 | 1 |
| 67 | 1 | 40.37260274 | 1 |
| 68 | 1 | 69.8630137 | 0 |
| 69 | 0 | 86.95890411 | 0 |
| 70 | 1 | 78.24657534 | 0 |
| 71 | 0 | 104.909589 | 0 |
| 72 | 1 | 77.39178082 | 0 |
| 73 | 1 | 94.29041096 | 0 |
| 74 | 1 | 38.69589041 | 1 |
| 75 | 1 | 16.60273973 | 1 |
| 76 | 1 | 7.989041096 | 1 |
| 77 | 0 | 18.90410959 | 1 |
| 78 | 0 | 74.56438356 | 0 |
| 79 | 1 | 62.23561644 | 0 |
| 80 | 1 | 46.81643836 | 0 |
| 81 | 1 | 9.89 | 1 |
| 82 | 1 | 189.08 | 0 |
| 83 | 1 | 36.44 | 1 |
| 84 | 0 | 106.02 | 0 |
| 85 | 1 | 11.86 | 1 |
| 86 | 1 | 0 | 1 |
| 87 | 1 | 39.29 | 0 |
| 88 | 1 | 21.82 | 1 |
| 89 | 0 | 116.57 | 0 |
| 90 | 1 | 36.73 | 0 |
| 91 | 1 | 11.24 | 1 |
| 92 | 1 | 10.94 | 1 |
| 93 | 1 | 80.66 | 0 |
| 94 | 1 | 97.08 | 0 |
| 95 | 1 | 32.03 | 1 |
| 96 | 1 | 94.55 | 0 |
| 97 | 0 | 36.96 | 1 |
| 98 | 0 | 125.77 | 0 |
| 99 | 0 | 129.41 | 0 |
| 100 | 0 | 131.71 | 0 |
| 101 | 0 | 120.54 | 0 |
| 102 | 0 | 55.2 | 1 |
| 103 | 1 | 22.37 | 1 |
| 104 | 1 | 42.48 | 0 |
| 105 | 0 | 85.13 | 0 |
| 106 | 0 | 113.77 | 0 |
| 107 | 1 | 9.2 | 1 |
| 108 | 0 | 109.93 | 0 |
| 109 | 0 | 24.05 | 1 |
| 110 | 1 | 110 | 0 |
| 111 | 1 | 62.29 | 0 |
| 112 | 0 | 101.52 | 0 |
| 113 | 0 | 107.3 | 0 |
| 114 | 1 | 60.78 | 0 |
| 115 | 1 | 17.45 | 1 |
| 116 | 1 | 49.68 | 0 |
| 117 | 1 | 48.92 | 0 |
| 118 | 0 | 95.41 | 0 |
| 119 | 1 | 96.16 | 0 |
| 120 | 1 | 45.08 | 0 |
| 121 | 0 | 5.49 | 0 |
| 122 | 1 | 67.88 | 0 |
| 123 | 1 | 83.98 | 0 |
| 124 | 1 | 62.03 | 0 |
| 125 | 1 | 58.35 | 0 |
| 126 | 0 | 47.15 | 0 |
| 127 | 0 | 66.17 | 0 |
| 128 | 1 | 0 | 1 |
| 129 | 1 | 13.4 | 1 |
| 130 | 1 | 52.47 | 0 |
| 131 | 1 | 57.33 | 0 |
| 132 | 0 | 51.84 | 0 |
| 133 | 1 | 35.75 | 0 |
| 134 | 1 | 33.64 | 1 |
| 135 | 0 | 28.39 | 0 |
| 136 | 1 | 19.4 | 1 |
| 137 | 1 | 30.03 | 1 |
| 138 | 0 | 30.42 | 1 |
| 139 | 0 | 22.57 | 1 |
| 140 | 1 | 93.17 | 0 |
| 141 | 0 | 17.71 | 1 |
| 142 | 0 | 3.35 | 1 |
| 143 | 1 | 36.04 | 1 |
| 144 | 0 | 62.36 | 1 |
| 145 | 0 | 49.41 | 0 |
| 146 | 0 | 54.7 | 0 |
| 147 | 0 | 14.06 | 0 |
| 148 | 0 | 54.47 | 0 |
| 149 | 1 | 55.16 | 0 |
| 150 | 0 | 41.82 | 0 |
| 151 | 1 | 35.22 | 0 |
| 152 | 1 | 37.52 | 0 |
| 153 | 0 | 37.75 | 0 |
| 154 | 0 | 35.65 | 0 |
| 155 | 1 | 30.78 | 0 |
| 156 | 0 | 26.28 | 0 |
| 157 | 0 | 27.14 | 0 |
| 158 | 1 | 14.03 | 1 |
| 159 | 0 | 22.83 | 0 |
| 160 | 1 | 7.39 | 0 |
| 161 | 0 | 8.11 | 0 |
| 162 | 1 | 7.75 | 0 |
| 163 | 1 | 12 | 1 |
| 164 | 0 | 11.47 | 0 |
| 165 | 0 | 7.69 | 0 |
| 166 | 1 | 93 | 0 |
| 167 | 1 | 37 | 0 |
| 168 | 1 | 31 | 0 |
| 169 | 1 | 120 | 0 |
| 170 | 1 | 25 | 1 |
| 171 | 1 | 21 | 1 |
| 172 | 1 | 45 | 0 |
| 173 | 1 | 45 | 1 |
| 174 | 1 | 52 | 0 |
| 175 | 1 | 16 | 1 |
| 176 | 1 | 70 | 0 |
| 177 | 1 | 77 | 0 |
| 178 | 1 | 47 | 0 |
| 179 | 1 | 11 | 1 |
| 180 | 1 | 76 | 0 |
| 181 | 1 | 69 | 0 |
| 182 | 1 | 28 | 0 |
| 183 | 1 | 34 | 0 |
| 184 | 1 | 10 | 1 |
| 185 | 1 | 29 | 0 |
| 186 | 1 | 36 | 0 |
| 187 | 1 | 31 | 0 |
| 188 | 1 | 36 | 0 |
| 189 | 1 | 6 | 1 |
| 190 | 1 | 33 | 0 |
| 191 | 1 | 10 | 1 |
| 192 | 1 | 27 | 0 |
| 193 | 1 | 45 | 0 |
| 194 | 1 | 6 | 1 |
| 195 | 1 | 67 | 0 |
| 196 | 1 | 11 | 1 |
| 197 | 1 | 51 | 0 |
| 198 | 1 | 48 | 0 |
| 199 | 1 | 58 | 0 |
| 200 | 1 | 3 | 1 |
| 201 | 1 | 48 | 0 |
| 202 | 1 | 10 | 1 |
| 203 | 1 | 120 | 0 |
| 204 | 1 | 74 | 1 |
| 205 | 1 | 104 | 0 |
| 206 | 1 | 22 | 1 |
| 207 | 1 | 53 | 0 |
| 208 | 1 | 37 | 1 |
| 209 | 1 | 11 | 1 |
| 210 | 1 | 39 | 1 |
| 211 | 1 | 45 | 0 |
| 212 | 1 | 45 | 0 |
| 213 | 1 | 23 | 1 |
| 214 | 1 | 34 | 1 |
| 215 | 1 | 15 | 1 |
| 216 | 1 | 51 | 0 |
| 217 | 1 | 44 | 0 |
| 218 | 1 | 30 | 0 |
| 219 | 1 | 27 | 0 |
| 220 | 1 | 26 | 0 |
| 221 | 1 | 25 | 0 |
| 222 | 1 | 10 | 1 |
| 223 | 1 | 23 | 0 |
| 224 | 1 | 23 | 0 |
| 225 | 1 | 23 | 0 |
| 226 | 1 | 24 | 0 |
| 227 | 0 | 56 | 0 |
| 228 | 1 | 66 | 1 |
| 229 | 1 | 29 | 1 |
| 230 | 1 | 129 | 0 |
| 231 | 1 | 9 | 1 |
| 232 | 1 | 195 | 0 |
| 233 | 0 | 193 | 0 |
| 234 | 1 | 109 | 0 |
| 235 | 0 | 107 | 0 |
| 236 | 1 | 13 | 1 |
| 237 | 0 | 30 | 1 |
| 238 | 0 | 13 | 1 |
| 239 | 0 | 106 | 0 |
| 240 | 1 | 43 | 1 |
| 241 | 0 | 87 | 0 |
| 242 | 1 | 77 | 0 |
| 243 | 1 | 109 | 0 |
| 244 | 1 | 14 | 1 |
| 245 | 0 | 96 | 0 |
| 246 | 1 | 87 | 0 |
| 247 | 0 | 97 | 0 |
| 248 | 1 | 26 | 1 |
| 249 | 1 | 16 | 1 |
| 250 | 0 | 86 | 0 |
| 251 | 1 | 104 | 0 |
| 252 | 1 | 70 | 0 |
| 253 | 0 | 85 | 0 |
| 254 | 0 | 16 | 1 |
| 255 | 0 | 11 | 1 |
